# Gene-alcohol interactions identify several novel blood pressure loci including a promising locus near *SLC16A9*

**DOI:** 10.3389/fgene.2013.00277

**Published:** 2013-12-12

**Authors:** Jeannette Simino, Yun Ju Sung, Rezart Kume, Karen Schwander, D. C. Rao

**Affiliations:** Division of Biostatistics, Washington University School of MedicineSt. Louis, MO, USA

**Keywords:** blood pressure, hypertension, alcohol, genome-wide, gene-alcohol interactions, gene-lifestyle interactions, interaction, GWAS

## Abstract

Alcohol consumption is a known risk factor for hypertension, with recent candidate studies implicating gene-alcohol interactions in blood pressure (BP) regulation. We used 6882 (predominantly) Caucasian participants aged 20–80 years from the Framingham SNP Health Association Resource (SHARe) to perform a genome-wide analysis of SNP-alcohol interactions on BP traits. We used a two-step approach in the ABEL suite to examine genetic interactions with three alcohol measures (ounces of alcohol consumed per week, drinks consumed per week, and the number of days drinking alcohol per week) on four BP traits [systolic (SBP), diastolic (DBP), mean arterial (MAP), and pulse (PP) pressure]. In the first step, we fit a linear mixed model of each BP trait onto age, sex, BMI, and antihypertensive medication while accounting for the phenotypic correlation among relatives. In the second step, we conducted 1 degree-of-freedom (df) score tests of the SNP main effect, alcohol main effect, and SNP-alcohol interaction using the maximum likelihood estimates (MLE) of the parameters from the first step. We then calculated the joint 2 df score test of the SNP main effect and SNP-alcohol interaction using MixABEL. The effect of SNP rs10826334 (near *SLC16A9*) on SBP was significantly modulated by both the number of alcoholic drinks and the ounces of alcohol consumed per week (*p*-values of 1.27E-08 and 3.92E-08, respectively). Each copy of the G-allele decreased SBP by 3.79 mmHg in those consuming 14 drinks per week vs. a 0.461 mmHg decrease in non-drinkers. Index SNPs in 20 other loci exhibited suggestive (*p*-value ≤ 1E-06) associations with BP traits by the 1 df interaction test or joint 2 df test, including 3 rare variants, one low-frequency variant, and SNPs near/in genes *ESRRG, FAM179A, CRIPT-SOCS5, KAT2B, ADCY2, GLI3, ZNF716, SLIT1, PDE3A, KERA-LUM, RNF219-AS1, CLEC3A, FBXO15,* and *IGSF5.* SNP-alcohol interactions may enhance discovery of novel variants with large effects that can be targeted with lifestyle modifications.

## Introduction

Hypertension afflicts 77.9 million adults in the United States (Go et al., 2013) and contributes to the public health burden of cardiovascular and cerebrovascular diseases (which cause death, induce functional disabilities, and reduce quality of life) (Roger et al., 2012). While increased awareness and treatment rates have partly alleviated the burden of hypertension, only half of all diagnosed hypertensives achieve their blood pressure (BP) goal (Roger et al., 2012). Dissecting the genetic and environmental architecture of BP regulation may inspire targeted lifestyle and pharmaceutical interventions that improve the prognosis and compliance of hypertensives. Although BP is a highly heritable trait, the identification of BP-associated genes has been slow and arduous compared to other complex traits like lipids. Genome-wide association studies (GWAS) of BP using up to 200,000 individuals have collectively identified ≈ 50 loci that explain less than 2.5% of the variance in BP (Adeyemo et al., 2009; Levy et al., 2009; Newton-Cheh et al., 2009; Padmanabhan et al., 2010; Ehret et al., 2011; Fox et al., 2011; Ho et al., 2011; Kato et al., 2011; Wain et al., 2011; Guo et al., 2012). The myriad of factors associated with BP, such as age, ethnicity, education, socioeconomic status, weight, physical activity, tobacco use, excessive alcohol consumption, psychosocial stress, and dietary factors (Xin et al., 2001; Go et al., 2013), complicate the dissection of its genetic underpinnings. These demographic and lifestyle factors may modulate the effect of genes on BP.

In this investigation, we focused on the role of alcohol consumption in the genetic and environmental architecture of BP. Alcohol consumption is a modifiable and highly prevalent behavior, as 51.5% of US adults consumed at least 12 alcoholic beverages in the past year (http://www.cdc.gov/nchs/fastats/alcohol.htm). Excessive alcohol consumption can be curbed to reduce the risk of hypertension (Fuchs et al., 2001; Kodavali and Townsend, 2006); a meta-analysis of 14 randomized clinical trials showed that reducing alcohol consumption in fairly heavy drinkers (>3 drinks per day) reduced systolic BP by 3.31 mmHg and diastolic BP by 2.04 mmHg (Xin et al., 2001). Yet, the effect of light-to-moderate alcohol consumption remains controversial (Klatsky and Gunderson, 2008; Leite et al., 2013) with mounting evidence that the genetic composition of an individual impacts the effect of alcohol consumption on hypertension risk. Candidate gene studies of hypertension and BP have implicated several interactions between alcohol and genes [*ADH2* (Sen Zhang et al., 2013), *ALDH2* (Chang et al., 2012; Nakagawa et al., 2013; Wang et al., 2013), *SOD2* (Nakagawa et al., 2013), *LEPR* (Sober et al., 2009), *ApoE* (Leite et al., 2013), *CYP11B2* (Pan et al., 2010), *NADH2* (Kokaze et al., 2004, 2007), *GNB3* (Polonikov et al., 2011), and *ADM* (Chen et al., 2013)].

Interactions between alcohol consumption and genes are biologically plausible, as the intermediate metabolites of alcohol can alter genes directly and influence their expression through epigenetic mechanisms (Alegria-Torres et al., 2011). The most common alcohol metabolism pathway involves two enzymes: alcohol dehydrogenase (ADH) and aldehyde dehydrogenase (ALDH). Ethanol is first oxidized to acetaldehyde by ADH, then the acetaldehyde is converted to acetate by ALDH, and the acetate is converted to water and carbon dioxide for elimination (National Institute on Alcohol Abuse and Alcoholism, 2007). Alcohol consumption can lead to acetaldehyde accumulation, which may be genotoxic (Joenje, 2011) and cause inter-strand crosslinking and other DNA damage (Lorenti Garcia et al., 2009; Joenje, 2011). Chronic alcohol consumption can lead to DNA hypomethylation through reductions in S-adenosylmethionine (Zakhari, 2013). Alcohol metabolism causes an increase in the NADH/NAD + ratio and generates reactive oxygen species and acetate, which can affect histone acetylation (Zakhari, 2013), damage DNA, and modify proteins (Finkel, 2011).

Most published GWAS ignore gene-alcohol interactions (Pan et al., 2011). Genome-wide studies incorporating gene-alcohol interactions may inform alcohol consumption guidelines, increase the accuracy of models predicting individual hypertension risk (Yi, 2010), enhance BP gene discovery efforts, and provide novel insights into the biological mechanisms and pathways underlying BP regulation (Thomas, 2010). Thus, we performed a genome-wide analysis of SNP-alcohol interactions on BP traits using 6882 (mostly) Caucasian participants from the Framingham SNP Health Association Resource (SHARe). We used participants 20–80 years old to examine the contribution of interactions between genetic variants and three alcohol measures (ounces of alcohol consumed per week, number of drinks consumed per week, and the number of days drinking alcohol per week) on four BP traits [systolic (SBP), diastolic (DBP), mean arterial (MAP), and pulse (PP) pressure]. Our aim was to identify novel BP loci with large interaction effects; discovery of such loci may facilitate alcohol intervention strategies and achievement of BP goals in genetically susceptible individuals, thereby reducing the public health burden of hypertension and its sequelae.

## Methods

### Subjects

We analyzed the Framingham SHARe data from dbGaP (accession number phs000007.v3.p2). The Framingham Heart Study (FHS) was initiated by the National Heart, Lung, and Blood Institute to investigate factors associated with the development of cardiovascular disease in a representative sample of the adult population of Framingham, Massachusetts (http://www.framinghamheartstudy.org/about-fhs/history.php). Our Framingham analysis set contained three inter-connected cohorts of mostly Caucasian participants: the Original cohort, the Offspring cohort, and the Third Generation (G3) Cohort. The Original Cohort, launched in 1948, included individuals aged 30–62 undergoing clinical examinations every 2 years (Dawber et al., 1951). The Offspring Cohort, launched in 1971, was formed from the biological descendents of the Original Cohort, as well as the spouses and offspring of the descendents (Feinleib et al., 1975). Following the baseline visit, participants in the Offspring Cohort underwent a second clinical visit 8 years later with subsequent visits every 4 years. The Third Generation Cohort, formed in 2002, included biological descendents or adopted offspring of the Offspring Cohort (Splansky et al., 2007). We analyzed a date-matched set of individuals aged 20–80 years old. The clinic visit dates from the twenty-sixth visit of the Original Cohort (May 1999 to November 2001) and the seventh visit of the Offspring Cohort (September 1998 to October 2001) were closest to each other, with the first visit of the Third Generation Cohort near the same time (April 2002 to July 2005); thus, we pooled the BP measurements from the twenty-sixth visit of the Original cohort (*N* = 6), the seventh visit of the Offspring Cohort (*N* ≈ 3000), and the first visit of the Third Generation Cohort (*N* ≈ 3800) to create a sample roughly analogous to a single-visit family study. Most members of the Original Cohort were beyond 80 years of age by the twenty-sixth exam. The six Original Cohort members that we included were part of extended pedigrees (one was a founder in a pedigree of 307 people). The average ages were 80, 61, and 40 years for the Original, Offspring, and Third Generation Cohorts, respectively.

### Phenotypes

In general, SBP and DBP were measured using a consistent protocol and a standard mercury column sphygmomanometer (portable Baumanometer 300 Model or wall-mounted Baumanometer E98169) in the clinic (the protocol descriptions are publicly available on dbGaP). Participants were seated for at least 5 min before the first BP measurement. Our analysis phenotype was the average of three BP measurements (one nurse/technician reading and two physician readings). MAP was estimated by the sum of two-thirds the (average) DBP and one-third of the (average) SBP. PP was computed as the difference between (average) SBP and (average) DBP. Any Original Cohort members unable to complete the twenty-sixth visit onsite had sitting or supine BP measured by a power aneroid sphygmomanometer (gauge only) offsite.

### Alcohol measures

We analyzed three alcohol measures: ounces of alcohol consumed per week, number of drinks consumed per week, and the number of days consuming alcohol per week. At each clinic visit, participants reported their consumption of beer, wine, and liquor/spirits through a standardized questionnaire (publicly available on dbGaP). A drink was defined as a typical serving size for each particular type of alcoholic beverage, such as a bottle of beer, a glass of wine, or a mixed drink or shot of liquor. The ounces of alcohol consumed per week was derived by Framingham researchers to depict the actual ethanol content consumed; only a certain percentage of an alcoholic beverage is ethanol and this varies by beverage type. We calculated the number of drinks per week by summing the number of drinks per week from each alcohol beverage category. The number of days drinking alcohol per week was a lower bound on their drinking activity: it equaled the maximum days per week an individual drank any one type of alcoholic beverage. Non-drinkers were assigned values of 0 for all alcohol measures collected and/or calculated in their cohort.

### Genotypes

SNPs were genotyped using the Affymetrix 500k array with the BRLMM calling algorithm and were filtered to remove Mendelian errors and gender discrepancies. Genotyped SNPs with call rates <90%, Hardy-Weinberg *p*-values < 1E-06, or less than 30 copies of the minor allele were excluded from our analysis. The dosage files for ~2.5 million imputed autosomal SNPs were available from the Framingham SHARe. We excluded SNPs with imputation quality *R*^2^ < 0.30 or less than 30 copies of the minor allele.

### Statistical analysis

We used the ABEL suite of packages to perform a two-step score test of the interaction between each SNP and alcohol measure on each BP trait (Aulchenko et al., 2007). In the first step, we fit a linear mixed model of each BP trait onto age, sex, BMI, and antihypertensive medication while accounting for the phenotypic correlation among relatives; this yielded maximum likelihood estimates (MLE) of the covariate coefficients, the residual variance, and the variance-covariance matrix for each BP trait (Aulchenko et al., 2007). In the second step, we conducted 1 degree-of-freedom (df) score tests of the SNP main effect, the alcohol main effect, and the SNP-alcohol interaction using the MLEs of the parameters from the first step (Aulchenko et al., 2010). We then employed MixABEL to calculate the joint 2 df score test of the SNP main effect and SNP-alcohol interaction. Since the 1 df interaction test statistics exhibited substantial inflation (genomic inflation factors up to 1.73), we applied the widely-accepted genomic control to achieve the expected distribution of *p*-values (Devlin and Roeder, 1999). The pre- and post-genomic control QQ plots are displayed in Figures S4–S7. For the joint 2 df test we did not perform any adjustment on the test statistics. Loci containing index SNPs with *p* ≤ 5E-08 were considered genome-wide significant, while loci harboring SNPs with *p* ≤ 1E-06 were deemed to have suggestive evidence of association with BP.

## Results

Our analysis sample included 6882 genotyped individuals with at least one BP measure, one alcohol measure, and non-missing values of all covariates. Table 1 displays the descriptive statistics for the Framingham subsample used in the interaction analysis of each alcohol measure. The ounces of alcohol consumed per week and the number of days drinking per week were not available in the Third Generation Cohort; thus the sample size for the number of alcoholic drinks per week, which was available in the Third Generation Cohort, was 2.27 times larger than that of the other alcohol measures. The percentage of alcohol drinkers was also higher for the number of drinks per week sample while the mean age (49 vs. 61 years), antihypertensive use (19 vs. 33%), and mean SBP (120.5 vs. 126.0 mmHg) were lower. Table 2 describes the pedigrees analyzed for each alcohol measure (Wigginton and Abecasis, 2005); the pedigree size ranged from 1 to 307 individuals in each of the 1166 families available for analysis. Although the sample size approximately doubled for the drinks per week analysis, the number of relative pairs increased many-fold due to the inclusion of all three interrelated cohorts.

**Table 1:** **Descriptive statistics for the BP traits, covariates, comorbidities, and alcohol measures**.

**Characteristics**	**Oz Alcohol/week**	**No.of days drink/week**	**No.of drinks/week**
Sample size	3027	3032	6882
% Male	46.38%	46.41%	46.69%
% Hypertensive	43.11%	43.14%	27.91%
% Taking antihypertensive meds	33.10%	33.11%	19.38%
% Taking cholesterol-lowering meds	20.68%	20.71%	13.34%
% Taking antidiabetic meds	6.48%	6.46%	3.91%
% Smoking regularly in the past year	13.68%	13.69%	15.78%
Age	60.77 ± 9.26	60.77 ± 9.26	49.26 ± 13.63
BMI	28.17 ± 5.30	28.17 ± 5.30	27.46 ± 5.47
SBP	125.95 ± 18.04	125.97 ± 18	120.52 ± 16.45
DBP	73.78 ± 9.36	73.78 ± 9.37	74.84 ± 9.37
MAP	91.16 ± 10.56	91.17 + 10.56	90.07 ± 10.35
PP	52.16 ± 15.76	52.17 + 15.77	49.26 ± 13.63
% Drinking (non-zero value of the specified alcohol measure)	66.34%	60.65%	74.43%
Mean of the alcohol measure specified by the column heading	2.61 ± 3.79	2.29 ± 2.65	5.46 ± 8.01
Range of the alcohol measure	0–38	0–7	0–101

Blood pressures, covariates, and alcohol measures are represented as the mean value ± the standard deviation.

**Table 2:** **Pedigree information for each analysis sample**.

**Characteristics**	**Oz Alcohol/week**	**No.of days drink/week**	**No.of drinks/week**
Sample Size from the Original/Offspring/Third Generation Cohort	6/3021/0	6/3026/0	6/3027/3849
Number of founders/non-founders	649/2378	650/2382	650/6232
Number of families	1040	1042	1166
Range of Pedigree Size	1–134	1–134	1–307
Parent-child pairs	32	32	4634
Full-sib pairs	1384	1386	5001
Half-sib pairs	63	63	277
Cousin-cousin pairs	798	800	7359
Grandparent-grandchild pairs	0	0	40
Avuncular pairs	153	153	5379

**Figure 1:**
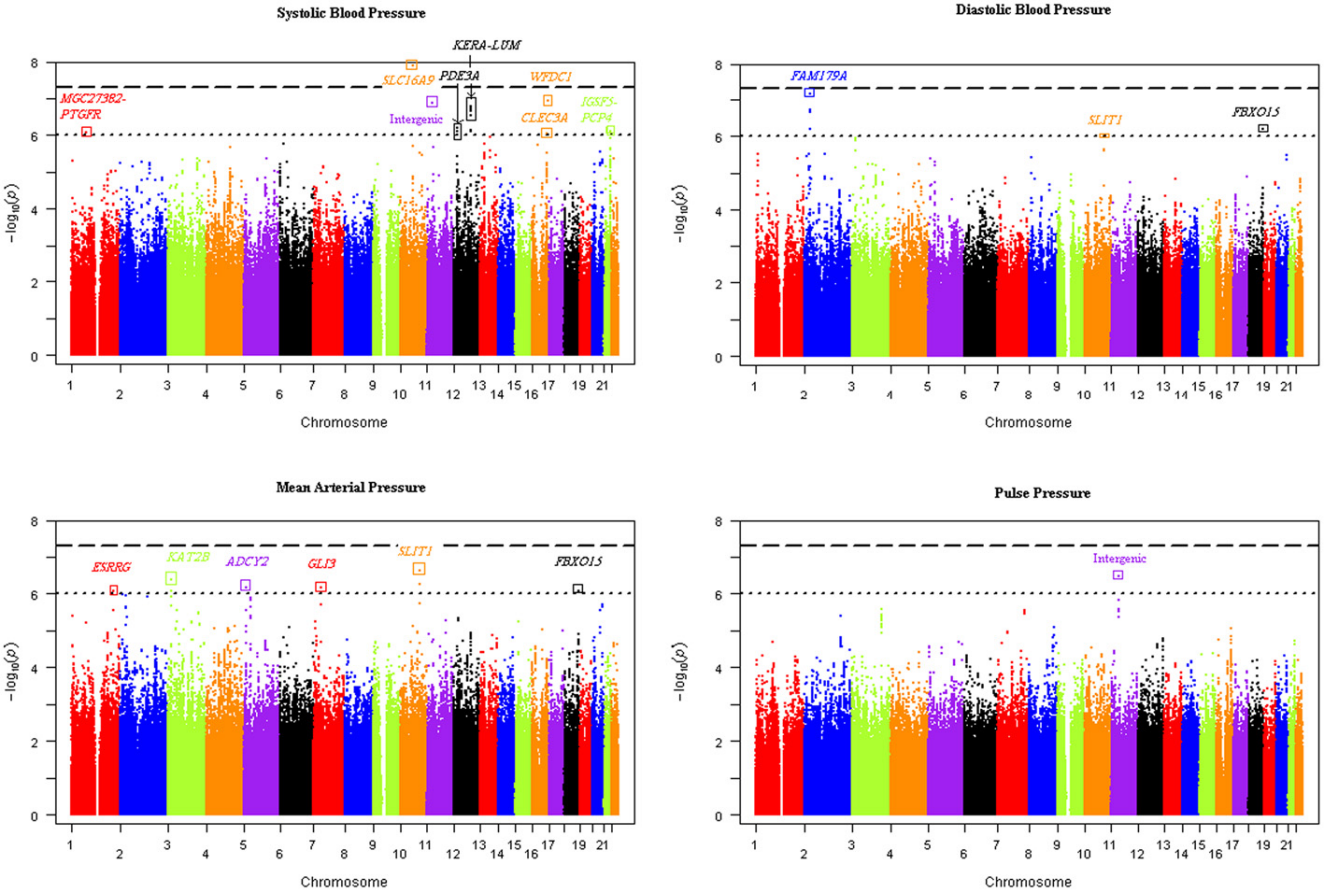
**Manhattan plots of the joint 2 degree-of-freedom test of the SNP main effect and SNP-drinks per week interaction for each blood pressure trait**. The -log(*p*-value) of the joint 2 df test of each SNP was plotted vs. the chromosomal location for all SNPs genome-wide. Sixteen unique loci were discovered using the four blood pressure traits.

Our genome-wide analysis of SNP × alcohol interactions yielded one significant (*p* ≤ 5E-08) and 20 suggestive (*p* ≤ 1E-06) BP loci (using either the 1 or 2 df test of SNP-alcohol interactions). The number of loci associated with SBP, DBP, MAP, and PP were 8, 4, 9, and 3, respectively; three loci were associated with more than one trait. The alcohol measure with the largest sample size, drinks of alcohol per week, enabled the discovery of 16 of the 21 loci. The Manhattan plots in Figure 1 display the genome-wide results for the joint 2 df test of the SNP main effect and SNP-drinks per week interaction on all 4 BP traits. Six loci achieved significant or suggestive associations for BP traits in the ounces of alcohol consumed per week interaction analysis, while only 3 loci reached suggestive association in the number of days drinking per week interaction analysis.

For each BP trait and alcohol measure, we selected an index SNP to represent each significant and suggestive locus. Association results for the index SNPs are displayed in Table 3. Index SNPs in 19 of the 21 loci exhibited interactions with alcohol as evidenced by either a suggestive 1 df interaction test or a significant/suggestive joint 2 df test in conjunction with a nominally significant (*p* < 0.05) 1 df interaction test; two PP loci (represented by rs4953404 and rs12292796) appeared to be driven by main effects only (1 df interaction tests have *p*-values > 0.3 as shown in Table 3) and will be excluded from further discussion (see Figures S8–S14 for the regional association plots for all the significant and suggestive loci). Twelve loci exhibited qualitative interactions by having opposite signs on the coefficients for the SNP main effect and the SNP × alcohol interaction. The coded alleles of these SNPs decreased BP for non-drinkers and increased BP for heavy-drinkers or vice versa (increased BP for non-drinkers and decreased BP for heavy drinkers). For these 12 loci, the SNP may have protective or harmful effects depending on alcohol consumption.

**Table 3:** **Index SNPs harbored in loci with significant and suggestive interactions by the one degree-of-freedom interaction test and the joint two degree-of-freedom test**.

**Chr**	**Position**	**SNPID**	**Genomic location**	**SNP type**	**BP trait**	**Alcohol measure**	**Ref allele**	**Coded allele freq**	**SNP main effect**		**SNP-alcohol interaction**		**1 df interaction test**	**2 df joint test**
									**β**	**SE(β )**	**β**	**SE(β )**		
1	78,659,796	rs648425	Intergenic *MGC27382-PTGFR*	Genotyped	SBP	Drinks/week	T	0.02	‒2.278	1.06	‒0.317	0.12	0.021	8.47E-07
1	214,823,444	rs17669622	Intron *ESRRG*	Imputed	MAP	Drinks/week	A	0.25	−0.174	0.245	−0.094	0.023	3.04E-04	8.63E-07
1	227,403,469	rs16849553	Intergenic near *RAB4A*	Genotyped	MAP	Oz alcohol/week	C	0.009	−5.758	1.482	1.452	0.254	1.35E-06	7.21E-07
2	29,101,501	rs13008299	Intron *FAM179A*	Genotyped	DBP	Drinks/week	G	0.29	−0.362	0.197	−0.068	0.018	7.36E-04	7.19E-08
2	46,739,646	rs4953404	Intergenic *CRIPT-SOCS5*	Imputed	PP	Days drinks/week	G	0.72	−0.138	0.03	0.006	0.009	0.502	7.85E-07
				Imputed	PP	Oz alcohol/week	G	0.72	−0.126	0.028	0.001	0.006	0.926	9.75E-07
3	20,076,567	rs9874923	Intron *KAT2B*	Genotyped	MAP	Drinks/week	T	0.43	−0.157	0.207	0.104	0.021	1.71E-05	4.18E-07
5	5,875,647	rs3852160	Intergenic	Imputed	MAP	Days drinks/week	C	0.39	−1.229	0.327	0.526	0.093	3.59E-07	1.65E-06
5	7,296,981	rs4537030	Intergenic near *ADCY2*	Imputed	MAP	Drinks/week	G	0.38	1.135	0.228	−0.087	0.021	2.12E-04	6.90E-07
7	42,351,145	rs7791745	Intergenic near *GLI3*	Imputed	MAP	Drinks/week	T	0.80	0.397	0.405	−0.196	0.039	1.17E-05	6.93E-07
7	57,587,798	rs11766519	Intergenic near *ZNF716*	Imputed	PP	Days drinks/week	C	0.13	0.312	0.065	−0.102	0.018	1.56E-07	1.12E-07
10	61,050,488	rs10826334	Intergenic near *SLC16A9*	Imputed	MAP	Oz alcohol/week	G	0.92	−0.092	0.535	−0.529	0.111	6.22E-05	9.29E-07
				Imputed	SBP	Oz alcohol/week	G	0.92	−0.647	0.79	−0.864	0.165	6.97E-05	**3.92E-08**
				Imputed	SBP	Drinks/week	G	0.91	−0.461	0.538	−0.238	0.052	6.57E-05	**1.27E-08**
10	98,784,049	rs12773465	Intron *SLIT1*	Imputed	MAP	Drinks/week	G	0.84	0.264	0.321	−0.159	0.031	7.33E-06	2.41E-07
10	98,799,693	rs7902871	Intron *SLIT1*	Imputed	DBP	Drinks/week	G	0.81	0.313	0.236	−0.12	0.023	2.78E-06	9.85E-07
11	23,911,889	rs7116456	Intergenic	Genotyped	SBP	Drinks/week	T	0.003	−7.284	2.649	0.929	0.167	1.08E-06	1.37E-07
11	39,382,675	rs12292796	Intergenic	Genotyped	PP	Drinks/week	C	0.07	0.173	0.034	−0.003	0.003	0.32	3.31E-07
12	20,490,379	rs10841530	Intron *PDE3A*	Imputed	SBP	Drinks/week	G	0.56	−1.02	0.298	0.155	0.029	4.37E-06	6.36E-07
12	89,998,553	rs991427	Intergenic *KERA-LUM*	Genotyped	SBP	Oz alcohol/week	T	0.14	1.96	0.614	0.412	0.138	0.023	1.60E-07
12	90,001,245	rs4494364	Intergenic *KERA-LUM*	Imputed	SBP	Drinks/week	A	0.14	0.868	0.436	0.142	0.043	0.004	1.66E-07
13	77,923,788	rs9318552	Intron RNF219-AS1	Imputed	DBP	Oz alcohol/week	T	0.79	−1.724	0.343	0.389	0.07	2.10E-07	5.33E-08
16	76,611,144	rs2735413	Upstream *CLEC3A*	Imputed	SBP	Drinks/week	A	0.31	−1.607	0.342	0.158	0.035	7.80E-05	9.18E-07
16	82,895,735	rs16963349	Intron *WFDC1*	Genotyped	SBP	Drinks/week	C	0.002	1.067	2.784	1.178	0.257	6.03E-05	1.14E-07
18	69,856,172	rs1943940	Intergenic near *FBXO15*	Imputed	DBP	Drinks/week	C	0.47	0.194	0.252	0.1	0.024	1.48E-04	6.16E-07
				Imputed	MAP	Drinks/week	C	0.47	0.248	0.284	0.106	0.027	4.48E-04	8.16E-07
21	40,101,946	rs2410182	Downstream *IGSF5*	Imputed	SBP	Oz alcohol/week	A	0.4	−0.245	0.439	−0.452	0.093	2.04E-04	6.87E-07
21	40,143,126	rs2837253	Intergenic *IGSF5-PCP4*	Imputed	SBP	Drinks/week	A	0.3	−0.777	0.349	0.182	0.035	5.10E-06	8.00E-07

The bolded p-values achieve genome-wide significance (*p* ≤ 5E-08).

**Figure 2:**
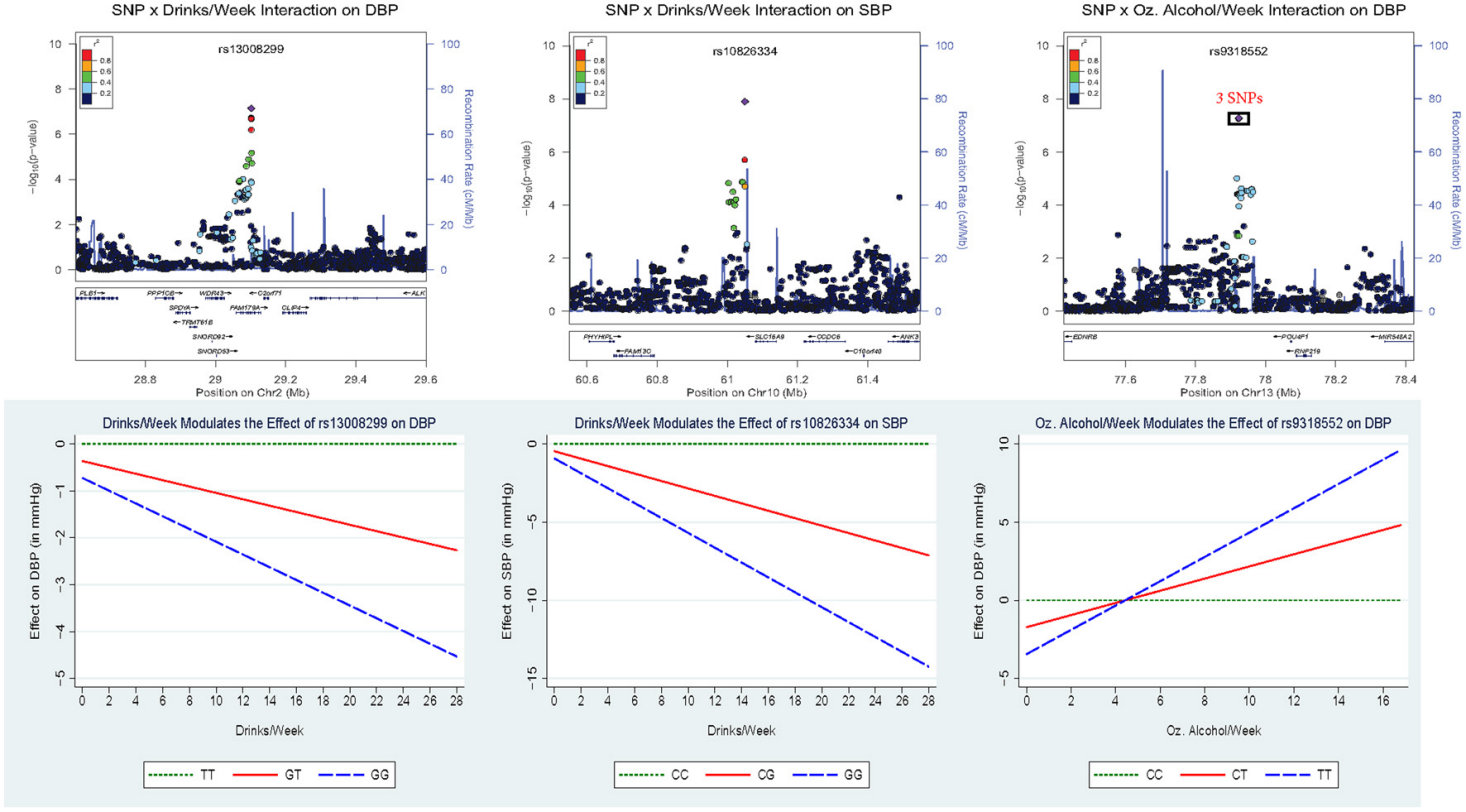
**Regional association plots for the three loci with the strongest statistical evidence, as well as plots of the effect of the index SNP as a function of alcohol consumption**. In the **top panel**, the *p*-value of the 2 df test is plotted vs. the chromosomal location (in basepairs) with the color scheme indicating the degree of linkage disequilibrium between each SNP and the index SNP (in purple). These regional plots were generated using LocusZoom (Pruim et al., 2010); rs9318552 was intronic to *RNF219-AS1* but this gene was omitted from the plot. The **bottom panel** depicts the estimated effect of the index SNP as a function of alcohol consumption.

Figure 2 displays the regional association plots for the three loci with the strongest statistical evidence, as well as plots of the effect of the index SNP as a function of alcohol consumption. SNP rs10826334 near monocarboxylic acid transporter 9 (*SLC16A9*) on chromosome 10 significantly interacted with both drinks per week and ounces of alcohol per week to influence SBP. Each copy of the G allele decreased SBP by 3.79 mmHg in individuals consuming 14 drinks per week as opposed to the 0.46 mmHg decrease per copy of the G allele in non-drinkers. Two other loci harbored index SNPs close to genome-wide significance. SNP rs9318552 intronic to RNF219 antisense RNA 1 (*RNF219-AS1*) on chromosome 13 interacted with ounces of alcohol per week to influence DBP (*p*-value = 5.3E-08). In non-drinkers, each copy of the T allele *decreased* DBP by 1.72 mmHg. However, in those drinking 8.4 ounces of alcohol per week (roughly the equivalent of 14 drinks) each copy of the T allele *increased* DBP by 1.54 mmHg. Thus, the T allele lowered DBP for alcohol consumption below 4.43 ounces of alcohol per week (~7.4 drinks per week) and raised DBP for alcohol consumption above this level. SNP rs13008299 intronic to family with sequence similarity 179, member A (*FAM179A*) on chromosome 2 interacted with drinks per week to influence DBP (*p*-value = 7.2E-08). Each copy of the G allele decreased DBP by 1.31 mmHg in individuals consuming 14 drinks per week as opposed to the 0.36 mmHg decrease per copy of the G allele in non-drinkers. All three of these common variants had supporting evidence in the region and non-negligible effect sizes as shown in Figure 2.

**Figure 3:**
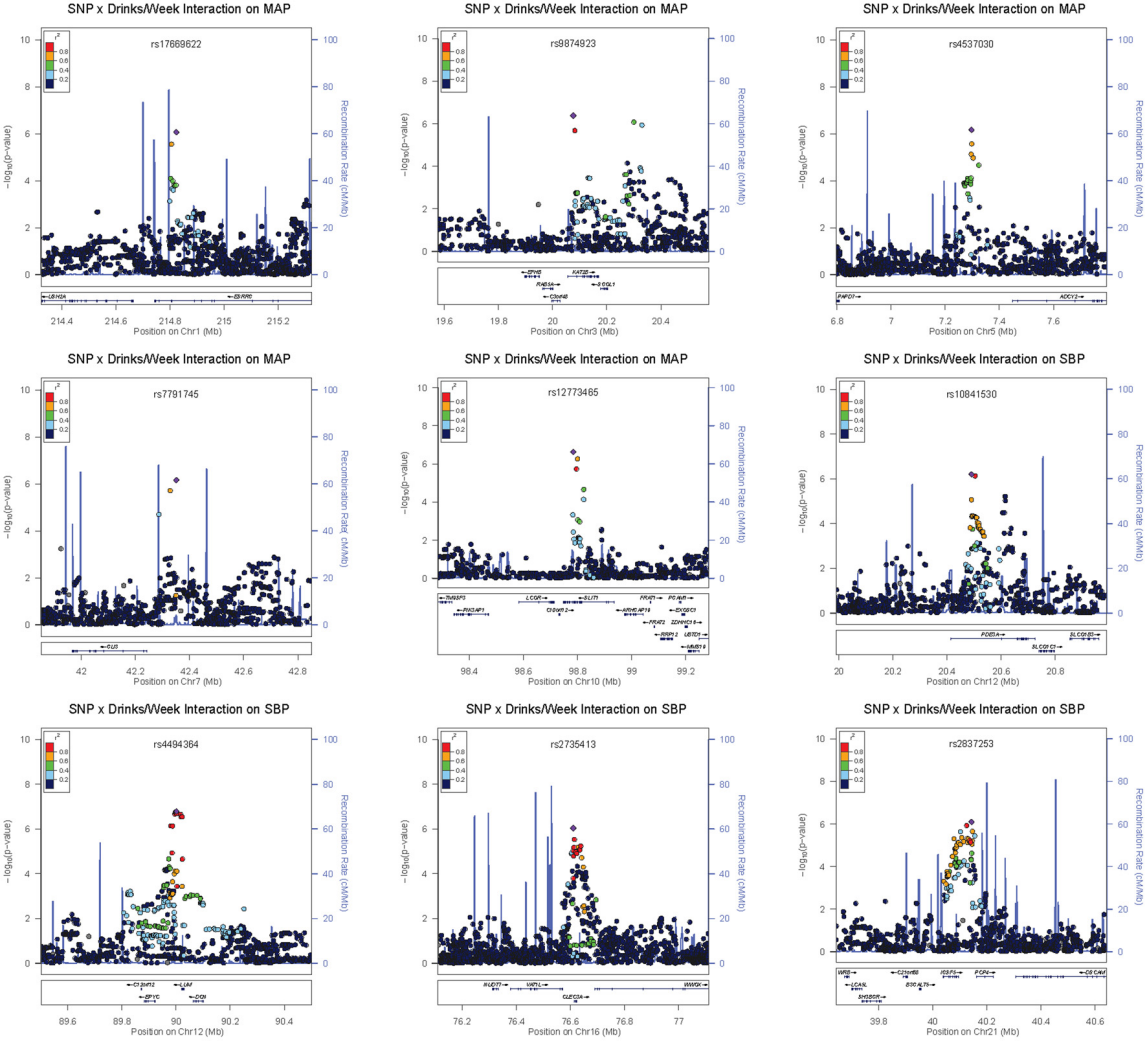
**Regional association plots for selected suggestive loci**.

Four loci contained low-frequency (rs648425) and rare (rs16849553, rs7116456, rs16963349) variants with suggestive alcohol interactions but these garnered no supporting evidence from common SNPs in the area (see the regional association plots in Figures S8–S14). As shown in Table 3, these SNPs had large estimated effects on BP but also had large standard errors for these estimates. Three of these were intergenic but SNP rs16963349 was intronic to WAP four-disulfide core domain 1 (*WFDC1*), a possible tumor suppressor gene. Common variants in the remaining suggestive loci were harbored in or near estrogen-related receptor gamma (*ESRRG*), K (lysine) acetyltransferase 2B (*KAT2B*), adenylate cyclase 2 (brain) (*ADCY2*), GLI family zinc finger 3 (*GLI3*), zinc finger protein 716 (*ZNF716*), slit homolog 1 (Drosophila) (*SLIT1*), phosphodiesterase 3A, cGMP-inhibited (*PDE3A*), keratocan (*KERA*)—lumican (*LUM*), C-type lectin domain family 3, member A (*CLEC3A*), F-box protein 15 (*FBXO15*), and immunoglobulin superfamily, member 5 (*IGSF5*). The regional association plots for selected suggestive loci are shown in Figure 3.

Table 4 displays published BP, cardiovascular, alcohol drinking, and homocysteine/methionine metabolism associations located within ≈500 kb of our index SNPs. These results were collated from the NHGRI GWAS catalog (http://www.genome.gov/gwastudies), the dbGaP database (http://www.ncbi.nlm.nih.gov/projects/gapplusprev/sgap_plus.htm), and the integrated map on the NCBI SNP website (http://www.ncbi.nlm.nih.gov/snp/) on November 4, 2013. Three of our loci (represented by SNPs rs648425, rs7791745, and rs16963349 in the *MGC27382-PTGFR, GLI3,* and *WFDC1* regions, respectively) were near significant (*p* ≤ 5E-08) BP associations identified in prior investigations of the Framingham Study (Levy et al., 2007). One additional locus (represented by two SNPs, rs12773465 and rs7902871, intronic to *SLIT1*) exhibited suggestive (*p* ≤ 1E-06) association in a prior Framingham analysis (Levy et al., 2007). SNP rs7791745 near *GLI3* appears to represent a novel locus; the *R*^2^ measure of linkage disequilibrium between our index SNP and the previously associated SNP was less than 0.2 (Johnson et al., 2008; Pruim et al., 2010). No linkage disequilibrium information was available for the other three loci to determine if these represented novel BP-associated loci (Johnson et al., 2008; Pruim et al., 2010).

**Table 4:** **Previous GWAS associations near our significant and suggestive loci**.

**Index SNP representing the significant or suggestive locus**			**Previous GWAS findings from the NHGRI GWAS catalog, the integrated map viewer in the SNP database, and dbGaP**					
**Chr**	**Position**	**Index SNP**	**Trait**	**SNP**	**Position (in bp)**	**Genomic location**	***P*-value**	**Database: PubMed ID referenced**
1	78,659,796	rs648425	Blood pressure	rs669733^†^	78,639,573	Intergenic *MGC27382-PTGFR*	**4E**-**08**	dbGaP: 17903302
			Blood pressure	rs10518590^†^	79,126,676	Downstream *ELTD1*	3E-06	dbGaP: 17903302
1	214,823,444	rs17669622	Cardiac hypertrophy	rs12757165	214,783,160	Intron *ESRRG*	1E-07	GWAS catalog: 21348951
1	227,403,469	rs16849553	Alcoholism	rs603404^†^	227,171,768	Intergenic *RHOU-ISCA1P2*	8E-05	dbGaP: None
			Blood pressure	rs237779^†^	227,501,899	Intron *RAB4A*	9E-05	dbGaP: None
2	29,101,501	rs13008299	Blood pressure	rs3190	28,878,983	UTR-3 *PP1CB*	9.9E-05	dbGaP: 17903302
2	46,739,646	rs4953404	Coronary heart disease	rs2346177	46,495,753	Intergenic *EPAS1*-*TMEM247*	2E-06	GWAS catalog: 21347282
3	20,076,567	rs9874923	Blood pressure	rs2203428	19,655,412	Intergenic *KCNH8-EFHB*	9E-05	dbGaP: None
			Coronary artery disease	rs1523351	20,552,477	Intergenic *SGOL1-VENTXP7*	6E-07	dbGaP: None
5	5,875,647	rs3852160	QT interval	rs7728043	5,950,694	Intergenic *KIAA0947*-*HMGB3P3*	1E-06	GWAS catalog: 20031603
			Hypertension	rs2937785	6,250,044	Intergenic *KIAA0947-FLJ33360*	9E-06	dbGaP: None
7	42,351,145	rs7791745	Blood pressure	rs949459	42,608,794	Intergenic *GLI3-TCP1P1*	7E-05	dbGaP: 17903302
			Blood pressure	rs10486744	42,861,079	Intergenic *TCP1P1-C7orf25*	**4E**-**09**	dbGaP: 17903302
10	61,050,488	rs10826334	Blood pressure	rs10509096	60,602,081	Upstream *PHYHIPL*	4E-06	dbGaP: 17903302
			Uric acid levels	rs12356193	61,083,359	Intron *SLC16A9*	**1E-08**	GWAS catalog: 19503597
			Carnitine	rs7094971	61,119,570	Intron *SLC16A9*	**3E-14**	GWAS catalog: 21886157
			Alcohol drinking	rs6479671	61,367,883	Intergenic *CCDC6-C10orf40*	**1E**-**11**	dbGaP: None
			Urate levels	rs1171614	61,139,544	UTR-5 *SLC16A9*	**2E-28**	GWAS catalog: 23263486
10	98,784,049	rs12773465	Cystathionine	rs3824789^*^	98,790,089	Intron *SLIT1*	6E-06	GWAS catalog: 23251661
	98,799,693	rs7902871	Total cysteine	rs12767760	99,284,787	Intron *UBTD1*	7E-06	GWAS catalog: 23251661
			Blood pressure	rs2275588^†^	99,331,175	Intron *ANKRD2*	3E-07	dbGaP: 17903302
11	23,911,889	rs7116456	Blood pressure	rs1519414^†^	23,624,092	Intergenic *RPS2P38-LUZP2*	4E-05	dbGaP: 17903302
			Echocardiography	rs10500968^†^	23,780,043	Intergenic *RPS2P38 LUZP2*	8E-07	dbGaP: 17903301
12	20,490,379	rs10841530	Baseline blood pressure	rs10841397	19,955,235	Intergenic *RPL34P25-TCP1P3*	9E-06	GWAS catalog: 22952603
			Aortic root size	rs10770612	20,121,906	Intron *LOC100506393*	**2E-08**	GWAS catalog: 19584346
			QT interval	rs1348582	20,423,023	Intron *PDE3A*	1E-06	GWAS catalog: 20031603
12	89,998,553	rs991427	Sudden cardiac arrest	rs10777317	90,504,505	Intergenic *DCN-BTG1*	5E-06	GWAS catalog: 21658281
	90,001,245	rs4494364						
16	76,611,144	rs2735413	Folate	rs4888671	76,341,081	Intergenic *NUDT7-VAT1L*	7E-06	GWAS catalog: 23251661
			Cardiac structure and function	rs2059238	76,816,311	Intron *WWOX*	3E-06	GWAS catalog: 19584346
			Cardiovascular disease	rs2278075^†^	76,848,674	Intron *WWOX*	**5E-09**	dbGaP: 17903304
16	82,895,735	rs16963349	Blood pressure	rs3096277^†^	82,321,705	Intron *CDH13*	**1E-09**	GWAS catalog: 17903302
18	69,856,172	rs1943940	Hypertension	rs4892138	69,609,765	Intergenic *NETO1-FBXO15*	1E-05	dbGaP: None
			Blood pressure	rs2115980	69,706,356	Intergenic *NETO1-FBXO15*	9.7E-05	dbGaP: None
21	40,101,946	rs2410182	Coronary heart disease	rs1735151	40,045,171	Intron *IGSF5*	9E-06	GWAS catalog: 21347282
	40,143,126	rs2837253	Blood pressure	rs2297263	40,377,724	Synonymous variant *DSCAM*	8E-05	dbGaP: 19060910
			Blood pressure	rs9977943	40,589,103	Intron *DSCAM*	9E-05	dbGaP: None

The R^2^ measure of linkage disequilibrium between the published association and our index SNP was determined using the SNAP database and the LocusZoom Plots when available.

† R^2^ values measuring linkage disequilibrium with the index SNPs were not available.

* SNP rs3824789 associated with cystathionine had R^2^ values of 0.305 and 0.448 with index SNPs rs12773465 and rs7902871, respectively. The remaining linkage disequilibrium values were less than 0.2. The bolded p-values achieve genome-wide significance (*p* ≤ 5E-08).

## Discussion

We identified one significant and twenty suggestive BP loci by exploiting gene-alcohol interactions in the analysis of 6882 participants from the FHS. Given that published GWAS with sample sizes up to 200,000 individuals have collectively identified fewer than 50 BP-associated loci, the identification of twenty-one candidate loci using interactions in a modest sized sample shows the promise of a more integrative gene and environment approach. Seventeen of these loci had failed to produce even suggestive evidence for BP in publicly-released findings from Framingham or other investigations. Our findings may also suggest why alcohol intake interventions may not reduce BP in all patients; some patients may be genetically susceptible to the effects of alcohol on BP and may experience the greatest BP changes with alcohol intake modifications, while others may have little genetic susceptibility and lack any marked BP response to alcohol consumption.

The interaction between alcohol and the significant locus near *SLC16A9* is biologically plausible. The index SNP (rs10826334) from this locus was 33 kb away from a variant significantly associated with uric acid (Kolz et al., 2009); uric acid has been associated with hypertension in humans and animal models (Mazzali et al., 2001; Feig, 2011). Diets rich in alcohol increase serum uric acid levels (Schlesinger, 2005) which may subsequently influence the renin-angiotensin system and reduce nitric oxide synthase in the macula densa of the kidney (Mazzali et al., 2001; Soltani et al., 2013), thereby influencing BP. As shown in Table 4, further support for this locus is provided by two previously reported associations; index SNP rs10826334 was located between SNP rs6479671 significantly associated with alcohol drinking (317 kb away; *p* = 1E-11; see dbGaP database) and SNP rs10509096 weakly (448 kb away; *p* = 4E-06) associated with BP in a previous investigation using the Framingham Study (Levy et al., 2007).

The gene-alcohol interactions identified in this investigation may provide insights into mechanisms underlying BP regulation and generate hypotheses to be tested in animal models. For index SNP rs10826334 in the significant locus near *SLC16A9*, the change from the C to G allele changes potential GATA, NERF1a, and PU.1 transcription-factor-binding motifs (Ward and Kellis, 2012). Rats fed an ethanol-containing liquid diet for 3 weeks exhibited increased mRNA and protein levels of PU.1 in the proximal tibia (Iitsuka et al., 2012). Therefore, one potential mechanism is that alcohol consumption increases levels of PU.1, fostering increased binding for individuals with the PU.1 motif, and altering the expression of an unknown downstream BP-associated gene.

We learned several lessons from this investigation. First, incorporating gene-environment interactions (GEI) enhanced the discovery of variants with large effect sizes. Even among non-drinkers, the estimated effect sizes of three rare/low-frequency variants ranged from 2.28 to 7.28 mmHg per copy of the coded allele. For individuals consuming 14 drinks per week (or ~8.4 ounces of alcohol), variants from eight loci had effect sizes exceeding 2 mmHg per copy of the coded allele. For significant SNP rs10826334, each copy of the G allele decreased SBP by 3.79 mmHg in individuals consuming 14 drinks per week. SNP rs16963349 had the largest effect increasing SBP by 17.56 mmHg for each copy of the rare C allele among consumers of 14 drinks per week. This SNP (intronic to *WFDC1*) is 574 kb from a known BP variant in the cadherin 13 (*CDH13*) gene (Levy et al., 2007). Such large effect sizes based on alcohol consumption have been previously reported. For 60 year-old non-smoking *apoE* ε 2 carriers in Brazil, Leite et al reported that drinkers had a mean SBP 16.5 mmHg higher than non-drinkers whereas among *apoE* ε 4 carriers there was no difference by drinking status (Leite et al., 2013). In general, our effect estimates for the significant and suggestive variants were consistent with those from other populations. In older Chinese men that consumed alcohol, BP traits varied by as much as 3.5 mmHg based on the genotypes at SNPs in *ADH2* and *ALDH2* (Sen Zhang et al., 2013). In Japanese men that drank alcohol daily, the effect of a variant in the *NADH2* gene was associated with a 4.77 mmHg increase in SBP (Kokaze et al., 2004). Thus, incorporating interactions between genes and environment in the analysis of sequence data may enhance effect sizes, and hence the statistical power, to discover BP variants from the whole allele frequency spectrum using reasonable sample sizes.

The second major lesson was that qualitative (“crossover”) interactions may be more pervasive than previously thought. We discovered twelve suggestive loci with interactions that changed direction of effect with alcohol consumption; in other words, alcohol consumption determined whether these variants increased or decreased BP. This further underscored the importance of incorporating GEI into genetic analyses. Omission of the SNP-alcohol interaction may mask the association between the genetic variant and BP (i.e., the effect of the SNP in drinkers may cancel out the effect of the SNP in non-drinkers) depending on the distribution of alcohol consumption in the sample. Perhaps part of the discordance of association results from previous BP studies can be attributed to differential distributions of environmental factors along with GEIs (Pan et al., 2010). For example, the *CYP11B2* variant shown to interact with alcohol by Pan et al. (2010) yielded inconsistent results in previous studies of hypertension; some studies reported that the T allele was associated with hypertension risk while others concluded that the C allele or no alleles were associated with hypertension (Pan et al., 2010). The discovery of qualitative interactions also implies that recommendations concerning alcohol consumption and hypertension risk may need to be personalized based on genotype (Taylor et al., 2009; Green and Guyer, 2011) or focused on high-risk genetic subgroups (Hunter, 2005; Murcray et al., 2009; Thomas, 2010). The incorporation of interactions may increase the accuracy of models predicting individual hypertension risk (Yi, 2010; Chang et al., 2012) and may increase the proportion of BP variance explained by current GWAS findings.

The third lesson was that we cannot discount the impact of the sample size and age distribution on the consistency of findings across alcohol measures. The sample size roughly doubled, and the average age decreased by 12 years, when the Third Generation cohort was added to the other two cohorts for the drinks per week analysis; this analysis identified 15 loci that appeared to interact with alcohol as evidenced by at least suggestive evidence for the 2 df test and nominal (*p*-value ≤ 0.05) significance for the 1 df interaction test. Of these 15 loci that interacted with the number of drinks consumed per week: three loci (represented by rs10826334, rs991427/rs4494364, and rs2410182/rs2837253) were also significant or suggestive for ounces of alcohol per week on the same BP trait; four loci (represented by rs13008299, rs7116456, rs2735413, and rs1943940) had *p*-values less than 1E-04 for at least one of the other two alcohol measures (with *p*-value ranks of 25–164 out of the roughly 2.5 million SNPs ordered smallest to largest for that alcohol measure); three loci (represented by rs648425, rs10841530, rs9874923) contained SNPs with *p*-values less than 1E-04 for a different alcohol measure in the 500 kilobase region to each side of the sentinel SNP; and one locus (represented by rs16963349) was so rare that association tests could not be performed using the smaller sample sizes from the ounces of alcohol consumed per week or the days drinking per week analyses.

There were a few limitations in this investigation. Alcohol consumption may have been underreported, a phenomenon common among excessive drinkers. The number of drinks per week, the alcohol measure available in the greatest number of participants, is imprecise due to the variability of ethanol content by alcoholic beverage type (beer, wine, hard liquor, etc.). Our analysis was largely restricted to individuals of European ancestry; race was not collected in the Original Cohort and 20% of the Offspring Cohort, thus race was missing for 9.8% of the 6882 individuals analyzed. In addition, one participant reported a non-Caucasian race in the ounces of alcohol consumed per week and days drinking alcohol per week subsamples, with 58 individuals (from 44 families that also contained 1146 Caucasian participants) reporting non-Caucasian races in the drinks per week sample. We analyzed all individuals but adjusted for population stratification through the genomic control method. Candidate gene studies in Japanese, Chinese, and Brazilian populations have reported gene-alcohol interactions (Kokaze et al., 2004, 2007; Pan et al., 2010; Chang et al., 2012; Chen et al., 2013; Leite et al., 2013; Nakagawa et al., 2013; Sen Zhang et al., 2013; Wang et al., 2013). Thus, by broadening the investigation of interactions between alcohol and genes to other ethnic groups, we may be able to capitalize on population-specific variants, different distributions of alcohol consumption, and different linkage disequilibrium patterns. We adjusted for antihypertensive use as a dichotomous yes/no predictor; the type of medication, as well as the genetic composition of an individual, may determine the treatment response. In our investigation, the 1 df interaction test suffered from substantial genomic inflation. The genomic control method we used to correct the 1 df interaction tests appeared to overcorrect the tail of the *p*-value distribution which should harbor true associations (see Figures S4–S7). Thus, we were overly conservative, potentially missing some real associations. Likewise, by not adjusting the 2 df test we may be reporting false positives.

Although we restricted this analysis to a single visit from each participant, we plan on following up with a longitudinal analysis of gene-alcohol interactions using the Framingham SHARe data. All our findings, both the cross-sectional and longitudinal, require further validation and replication in an independent sample. Lastly, we did not account for genetic variants influencing alcohol metabolism (particularly *ADH2*, *ALDH2*). Different variants of the ADH and ALDH enzymes work at different efficiencies, thereby determining the concentration of intermediate metabolites such as acetaldehyde in the body following a given amount of ethanol consumption. A slow ALDH enzyme can cause acetaldehyde accumulation compared to the wild-type ALDH variant (National Institute on Alcohol Abuse and Alcoholism, 2007). Thus, by testing SNP-alcohol interactions in individuals with and without the slow ALDH variants, we can test whether the interaction effects of the novel variants are larger when acetaldehyde accumulates. This may help refine the metabolite or mechanism through which the alcohol interactions are occurring.

In summary, reliance on genetic main effects may impede discovery of novel variants with large effects that can be targeted with lifestyle modifications. GEIs have to potential to increase the clinical translatability of genetic findings and elucidate mechanisms underlying BP regulation.

## Author contributions

Concept and design (Jeannette Simino, Yun Ju Sung, D. C. Rao), acquisition from dbGaP and assembly of analysis data (Karen Schwander), data analysis (Rezart Kume and Yun Ju Sung), interpretation of results (all authors), manuscript writing (Jeannette Simino, Rezart Kume), and final review and approval of the manuscript (all authors).

### Conflict of interest statement

The authors declare that the research was conducted in the absence of any commercial or financial relationships that could be construed as a potential conflict of interest.

## Supplementary material

Figure S4 **Quantile-quantile plots of the 1 df SNP-alcohol interaction tests for SBP**. Plots are presented for all three alcohol measures (drinks per week, days drinking per week, and ounces of alcohol per week) before and after the genomic control adjustment.

Figure S5 **Quantile-quantile plots of the 1 df SNP-alcohol interaction tests for DBP**. Plots are presented for all three alcohol measures (drinks per week, days drinking per week, and ounces of alcohol per week) before and after the genomic control adjustment.

Figure S6 **Quantile-quantile plots of the 1 df SNP-alcohol interaction tests for MAP**. Plots are presented for all three alcohol measures (drinks per week, days drinking per week, and ounces of alcohol per week) before and after the genomic control adjustment.

Figure S7 **Quantile-quantile plots of the 1 df SNP-alcohol interaction tests for PP**. Plots are presented for all three alcohol measures (drinks per week, days drinking per week, and ounces of alcohol per week) before and after the genomic control adjustment.

Figure S8–S14 **Regional association plots for loci with suggestive or significant associations by the 1 df interaction test or the joint 2 df test**. Plots are ordered by chromosome and position, with symbol color indicating the strength of the linkage disequilibrium (r-squared value) between the plotted SNP and the index SNP.
