# Supplementary figures and images for: Gene-alcohol interactions identify several novel blood pressure loci including a promising locus near SLC16A9
Source: Front Genet. 2013 Dec 12;4:277. doi: 10.3389/fgene.2013.00277 (PMC3860258; doi:10.3389/fgene.2013.00277)

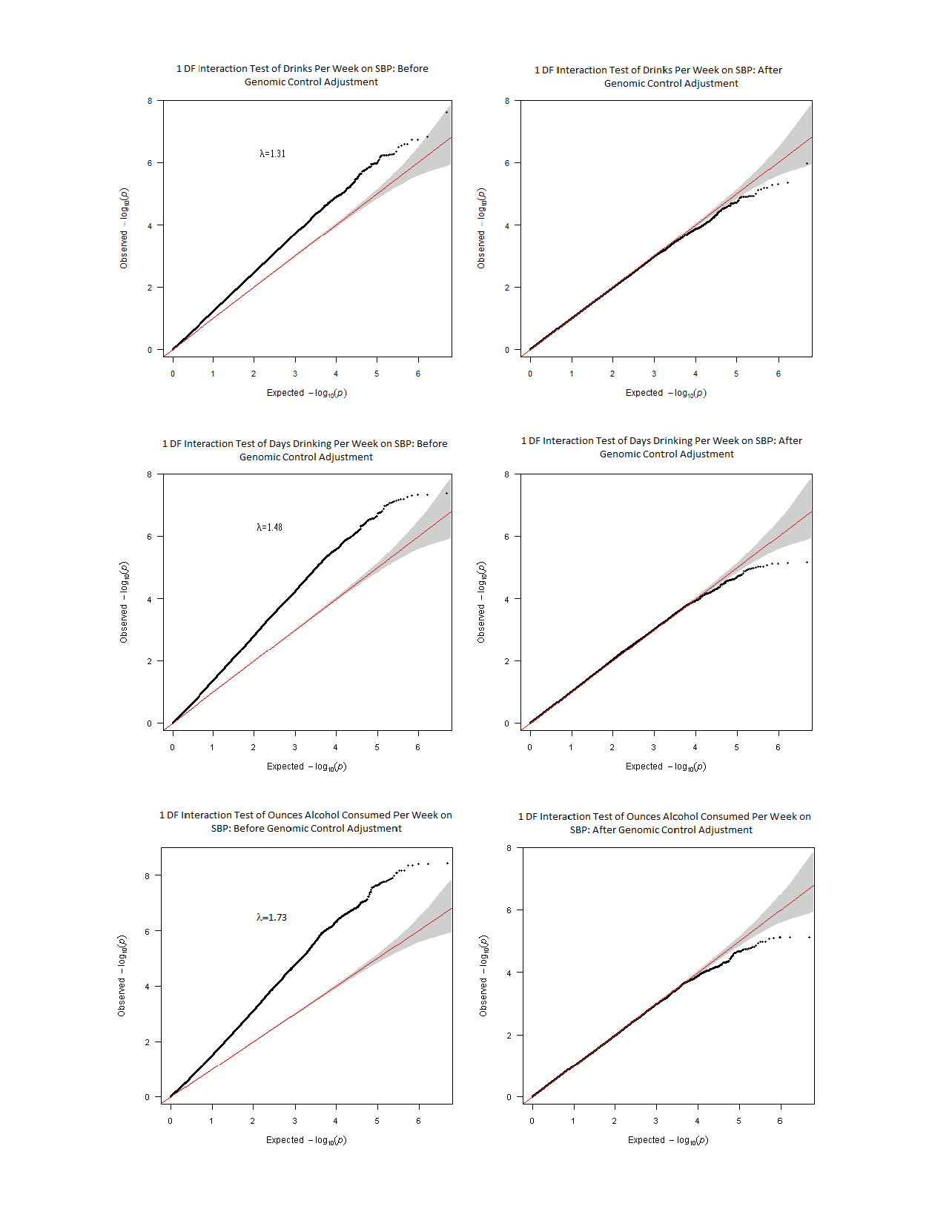

Supplement: Figure S4 — Quantile-quantile plots of the 1 df SNP-alcohol interaction tests for SBP. Plots are presented for all three alcohol measures (drinks per week, days drinking per week, and ounces of alcohol per week) before and after the genomic control adjustment. [file DataSheet1.ZIP › 66965_Simino_Figure_4.TIFF]

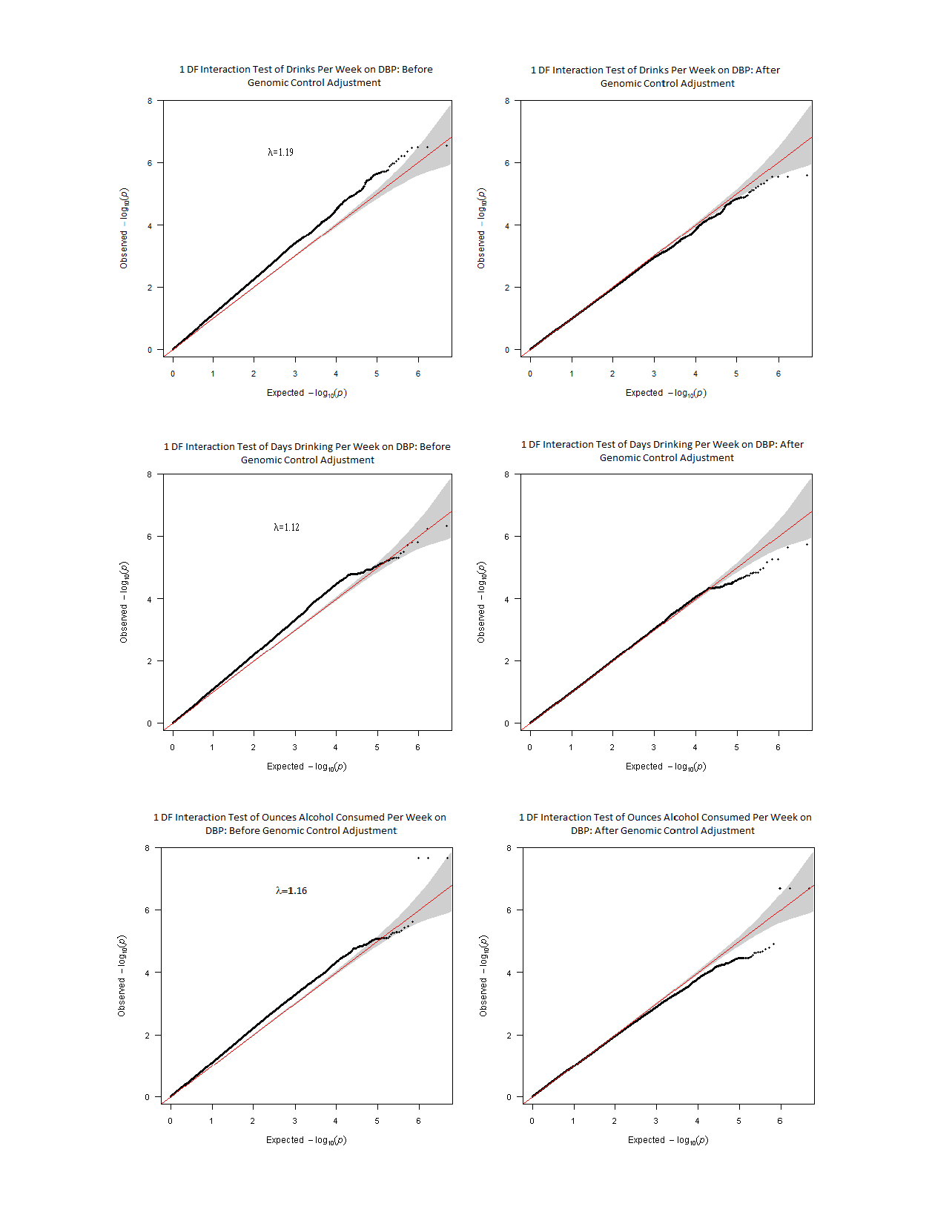

Supplement: Figure S4 — Quantile-quantile plots of the 1 df SNP-alcohol interaction tests for SBP. Plots are presented for all three alcohol measures (drinks per week, days drinking per week, and ounces of alcohol per week) before and after the genomic control adjustment. [file DataSheet1.ZIP › 66965_Simino_Figure_5.TIFF]

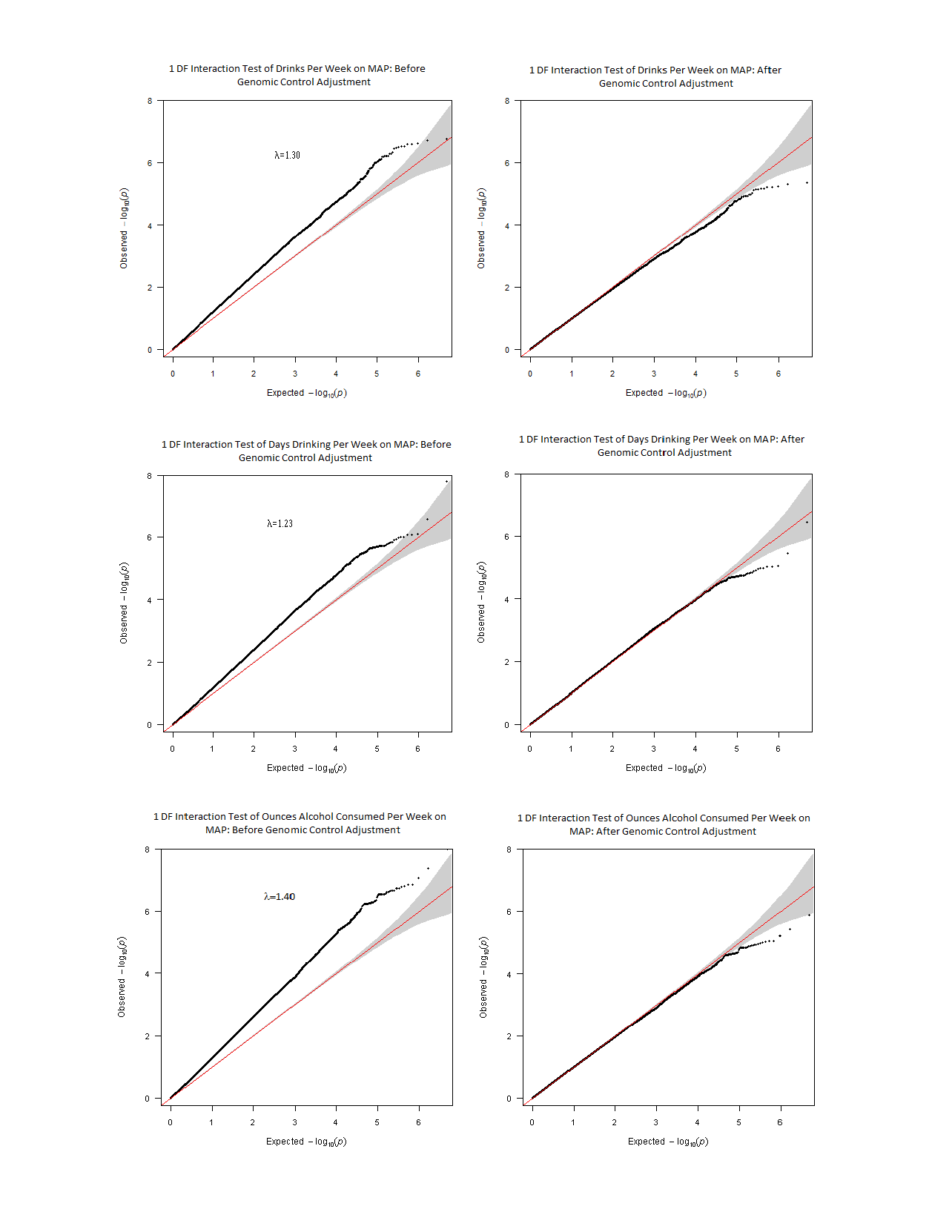

Supplement: Figure S4 — Quantile-quantile plots of the 1 df SNP-alcohol interaction tests for SBP. Plots are presented for all three alcohol measures (drinks per week, days drinking per week, and ounces of alcohol per week) before and after the genomic control adjustment. [file DataSheet1.ZIP › 66965_Simino_Figure_6.TIFF]

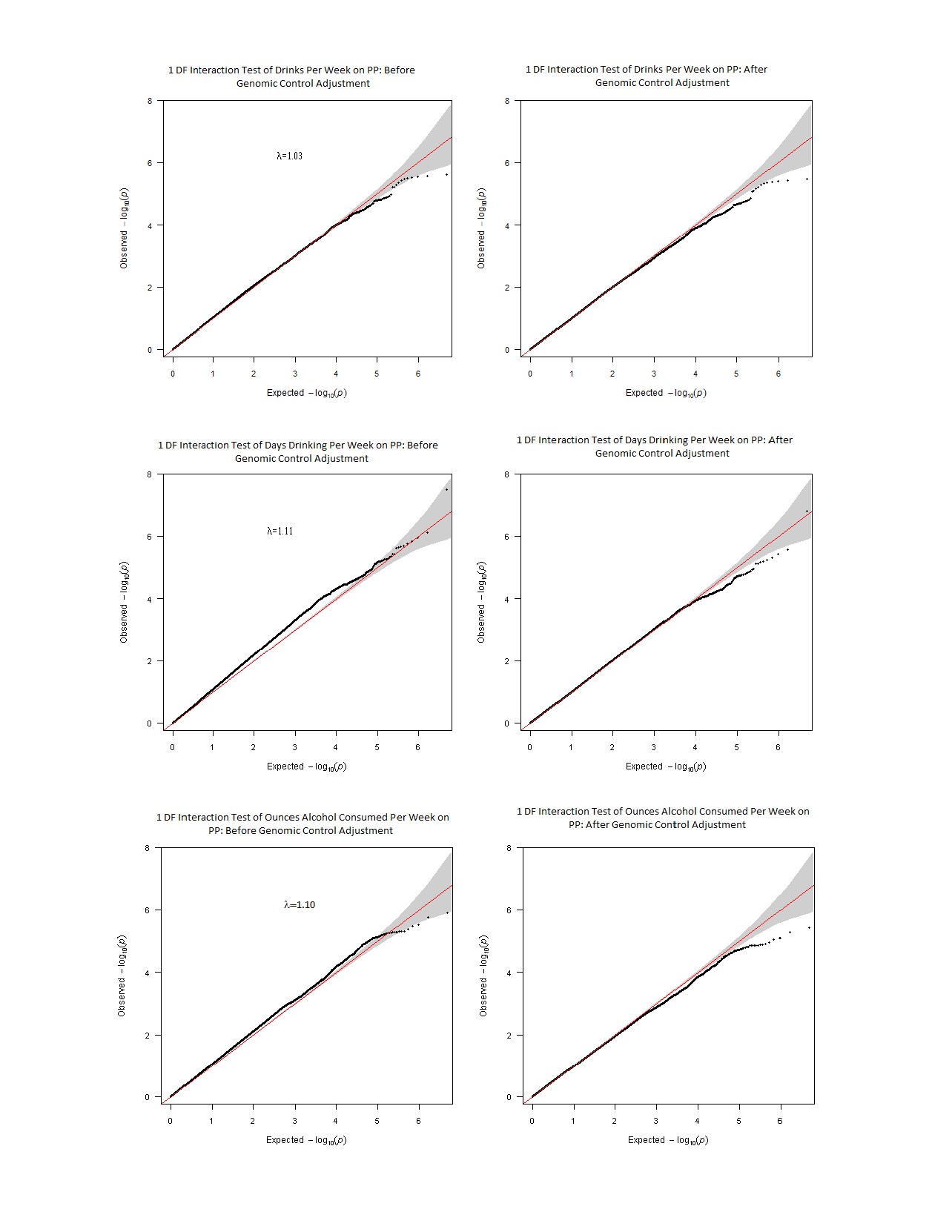

Supplement: Figure S4 — Quantile-quantile plots of the 1 df SNP-alcohol interaction tests for SBP. Plots are presented for all three alcohol measures (drinks per week, days drinking per week, and ounces of alcohol per week) before and after the genomic control adjustment. [file DataSheet1.ZIP › 66965_Simino_Figure_7.TIFF]

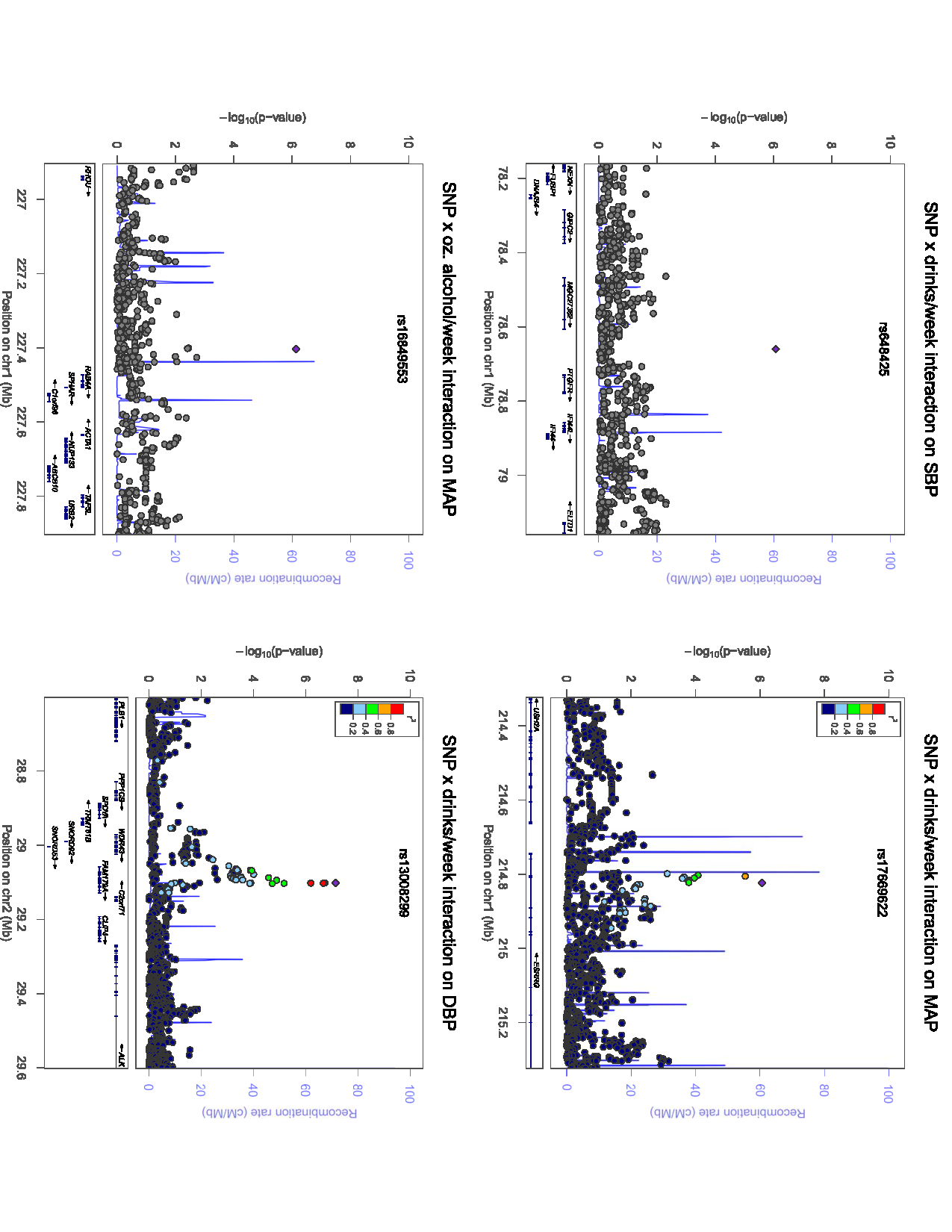

Supplement: Figure S4 — Quantile-quantile plots of the 1 df SNP-alcohol interaction tests for SBP. Plots are presented for all three alcohol measures (drinks per week, days drinking per week, and ounces of alcohol per week) before and after the genomic control adjustment. [file DataSheet1.ZIP › 66965_Simino_Figure_8.TIF]

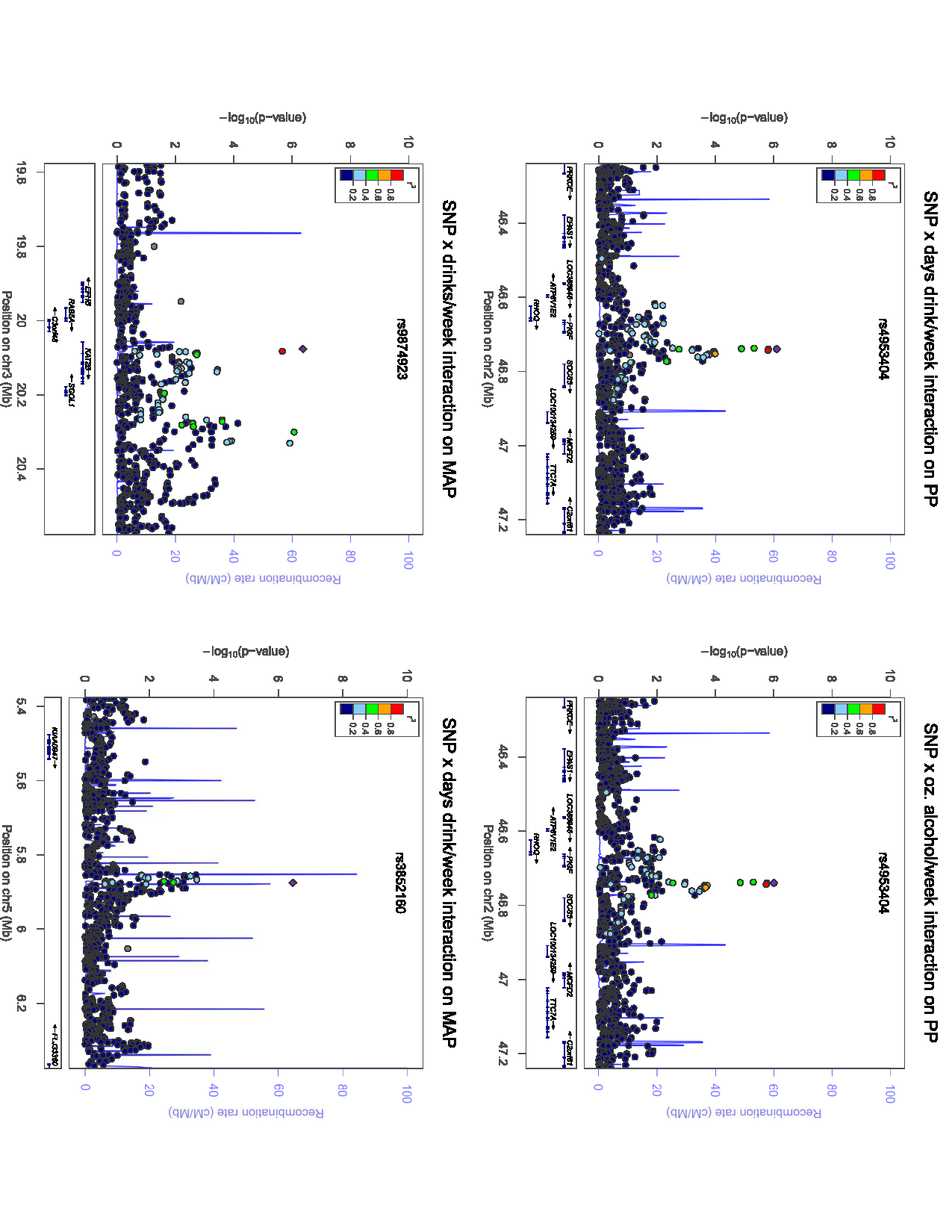

Supplement: Figure S4 — Quantile-quantile plots of the 1 df SNP-alcohol interaction tests for SBP. Plots are presented for all three alcohol measures (drinks per week, days drinking per week, and ounces of alcohol per week) before and after the genomic control adjustment. [file DataSheet1.ZIP › 66965_Simino_Figure_9.TIF]

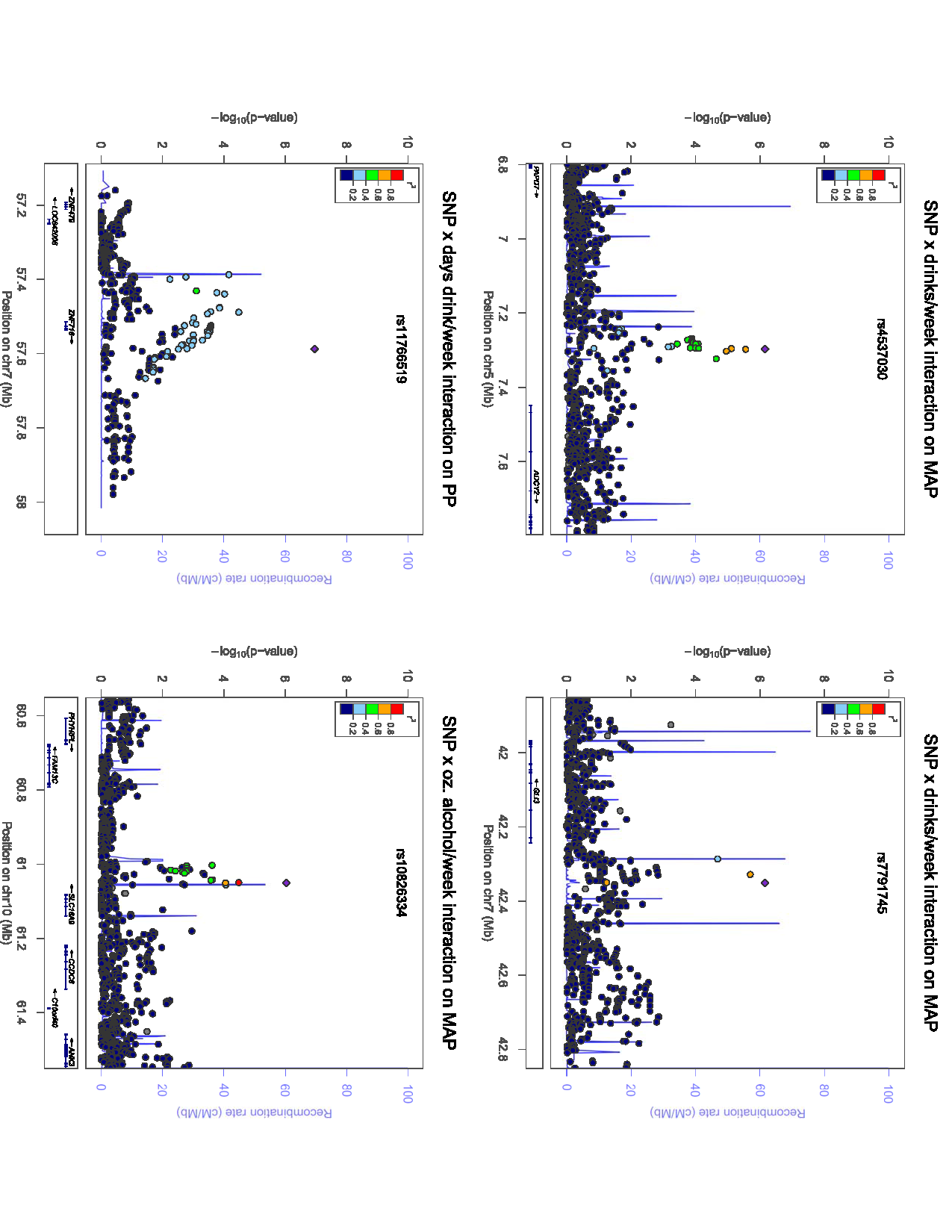

Supplement: Figure S4 — Quantile-quantile plots of the 1 df SNP-alcohol interaction tests for SBP. Plots are presented for all three alcohol measures (drinks per week, days drinking per week, and ounces of alcohol per week) before and after the genomic control adjustment. [file DataSheet1.ZIP › 66965_Simino_Figure_10.TIF]

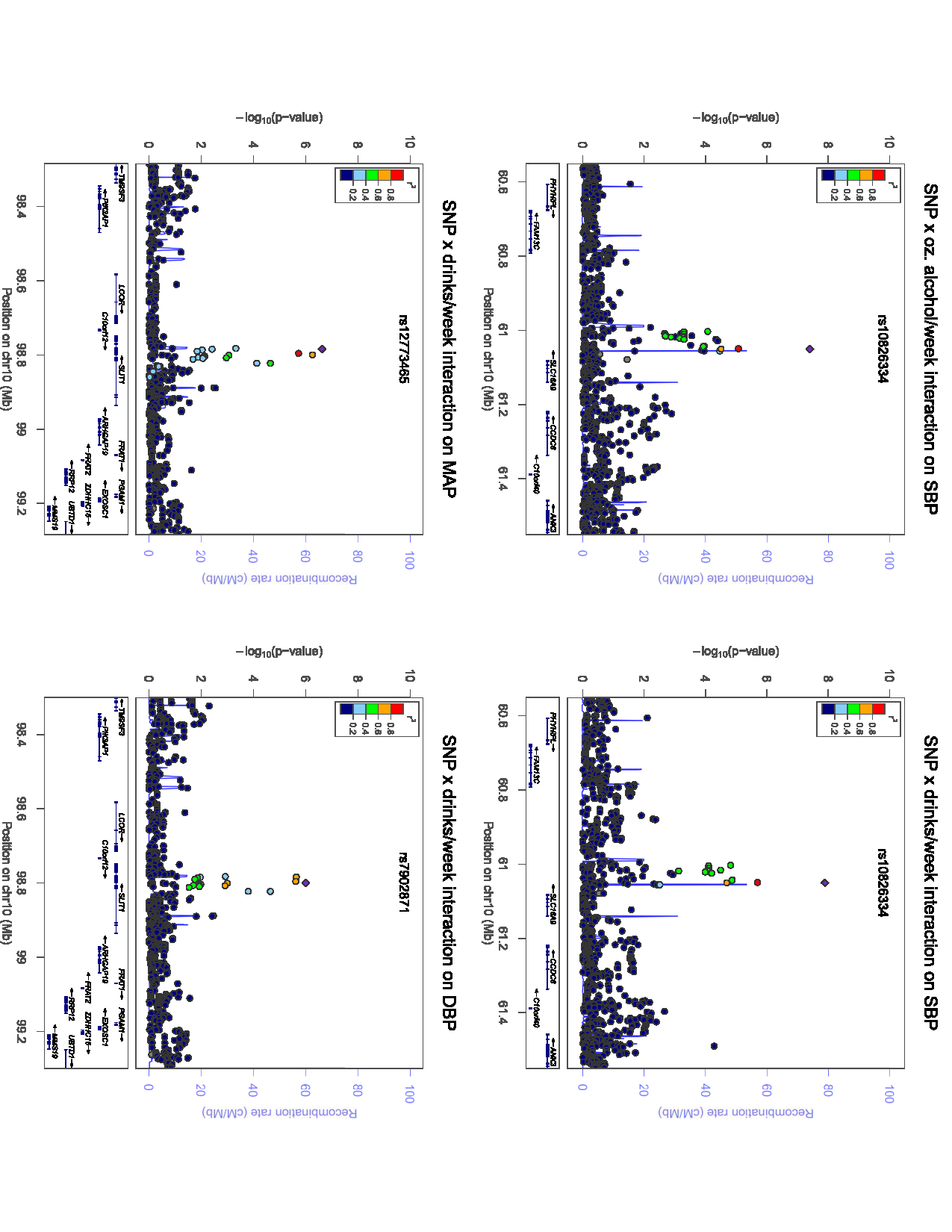

Supplement: Figure S4 — Quantile-quantile plots of the 1 df SNP-alcohol interaction tests for SBP. Plots are presented for all three alcohol measures (drinks per week, days drinking per week, and ounces of alcohol per week) before and after the genomic control adjustment. [file DataSheet1.ZIP › 66965_Simino_Figure_11.TIF]

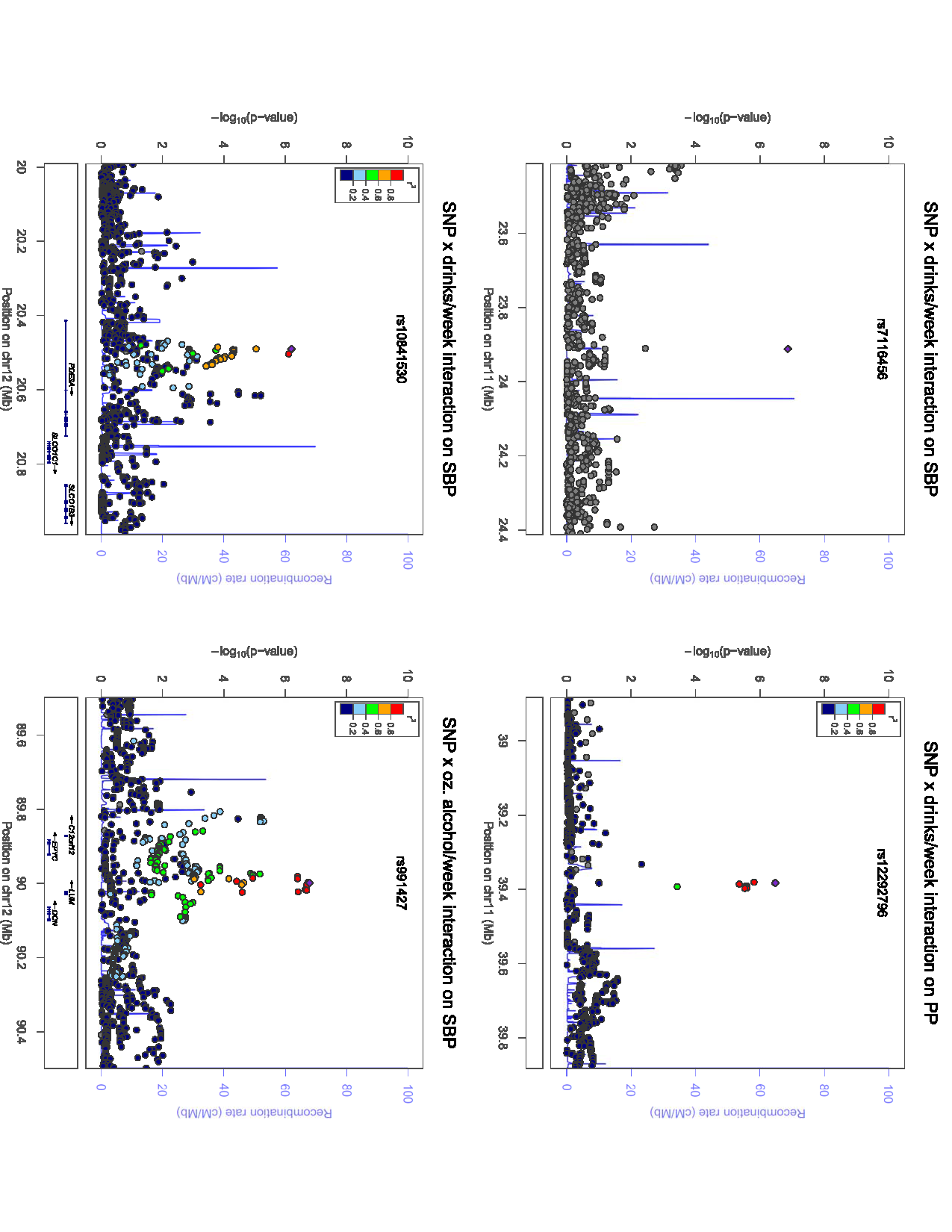

Supplement: Figure S4 — Quantile-quantile plots of the 1 df SNP-alcohol interaction tests for SBP. Plots are presented for all three alcohol measures (drinks per week, days drinking per week, and ounces of alcohol per week) before and after the genomic control adjustment. [file DataSheet1.ZIP › 66965_Simino_Figure_12.TIF]

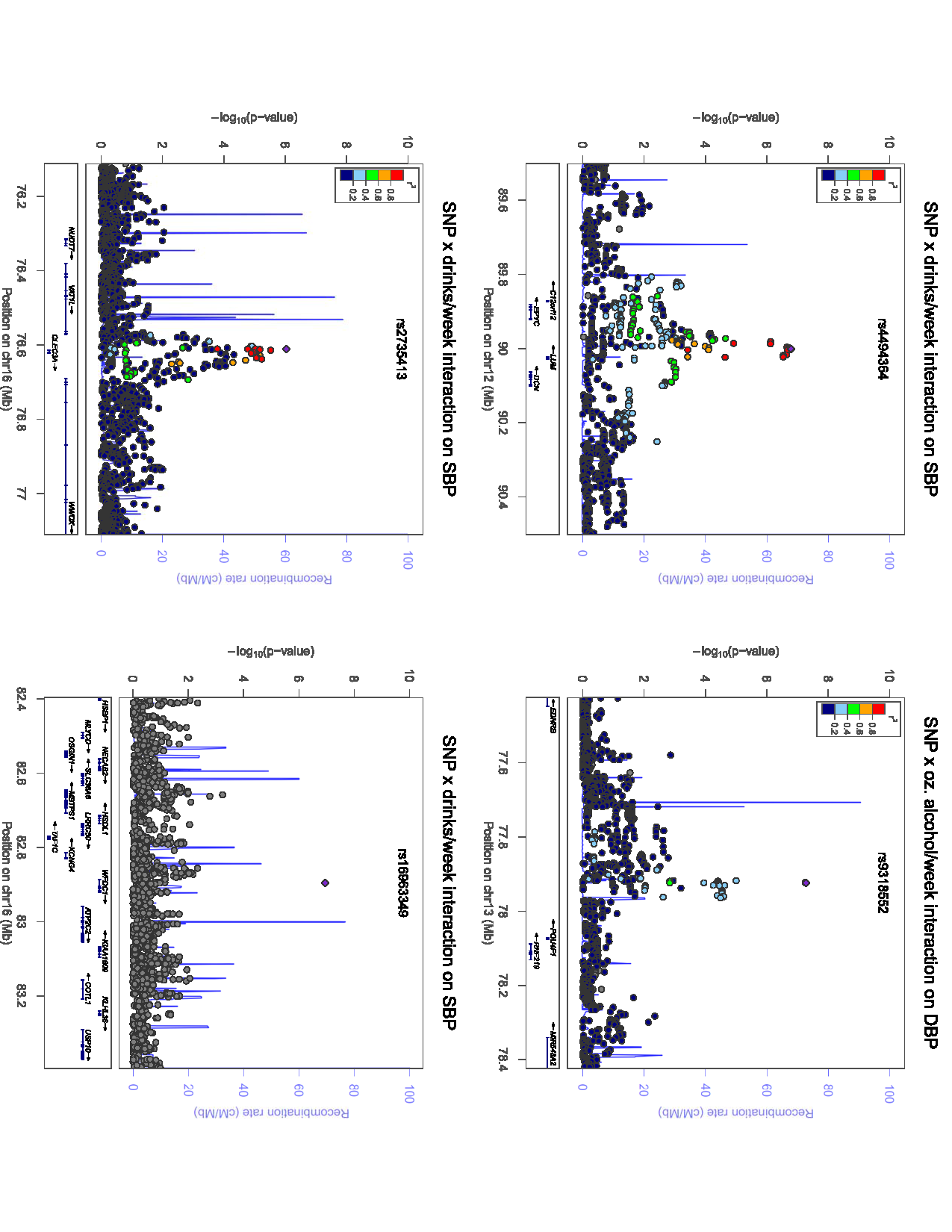

Supplement: Figure S4 — Quantile-quantile plots of the 1 df SNP-alcohol interaction tests for SBP. Plots are presented for all three alcohol measures (drinks per week, days drinking per week, and ounces of alcohol per week) before and after the genomic control adjustment. [file DataSheet1.ZIP › 66965_Simino_Figure_13.TIF]

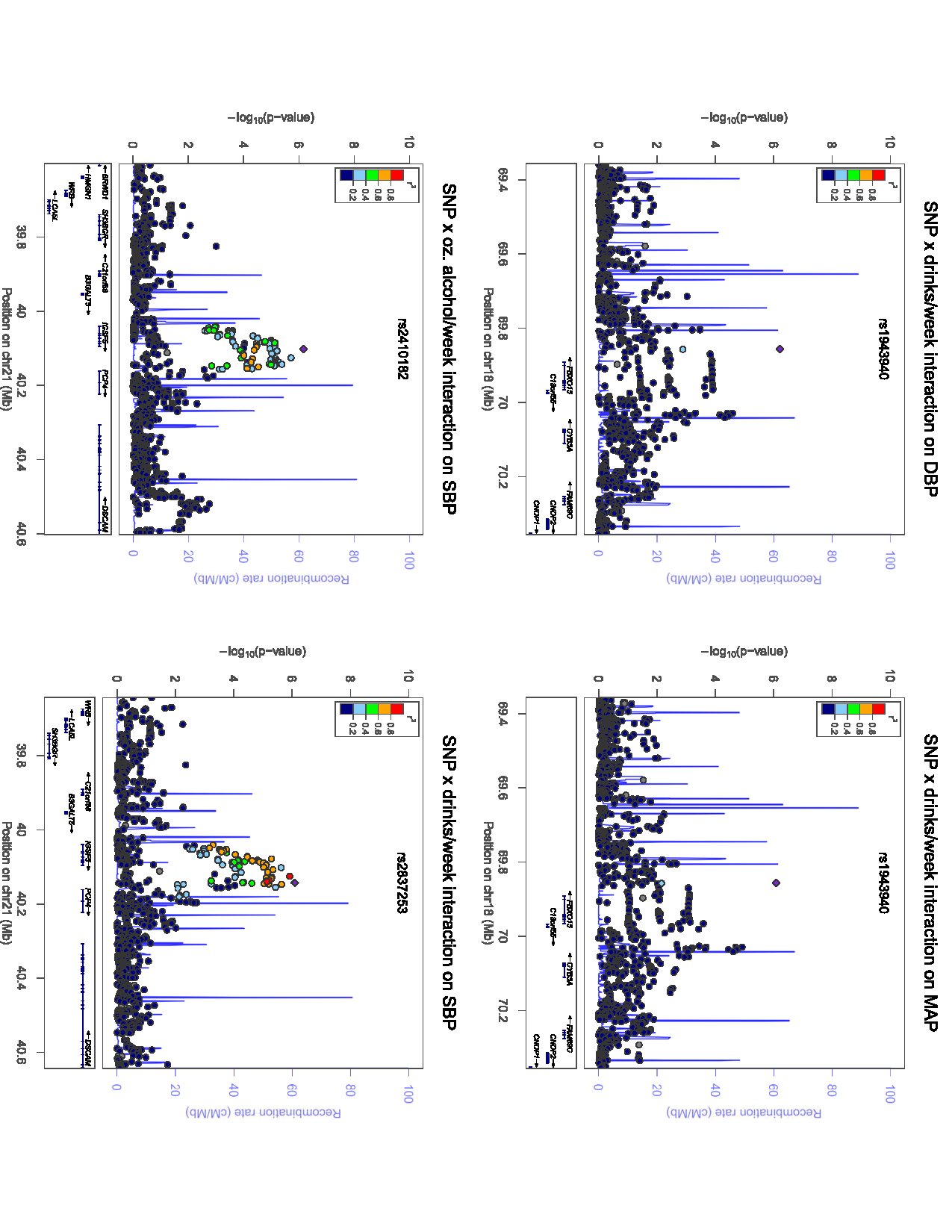

Supplement: Figure S4 — Quantile-quantile plots of the 1 df SNP-alcohol interaction tests for SBP. Plots are presented for all three alcohol measures (drinks per week, days drinking per week, and ounces of alcohol per week) before and after the genomic control adjustment. [file DataSheet1.ZIP › 66965_Simino_Figure_14.TIF]
